# Structural Fluctuations at Nanoscale and Cooperative Molecular Dynamics in Bulk Water

**DOI:** 10.1021/acs.jpclett.5c00735

**Published:** 2025-06-04

**Authors:** Margarita Russina, Gerrit Günther, Bela Farago, Earl Babcock, Zahir Salhi, Alexander Ioffe, Ferenc Mezei

**Affiliations:** † Helmholtz-Zentrum Berlin, Hahn-Meitner Platz 1, 14109 Berlin, Germany; ‡ Institute Laue-Langevin, 71 avenue des Martyrs CS 20156, 38042 Grenoble Cedex 9, France; § Jülich Centre for Neutron Science (JCNS) at Heinz Maier-Leibnitz Zentrum (MLZ), Forschungszentrum Julich GmbH, 85747 Garching, Germany; ∥ KFKI Campus, Konkoly Thege u 29-33, H-1525 Budapest, Hungary

## Abstract

The investigation of cooperative dynamics in H_2_O, visible in coherent neutron scattering, has been hindered until now due to the very small signal. Using neutron polarization analysis, we were able, for the first time, to directly measure the coherent neutron scattering signal in light water with unprecedented accuracy. The observed coherent signal is enhanced in the intermediate *Q* range of 0.2 to 1 Å^–1^, providing clear evidence that intermolecular interactions in water extend beyond the distances between nearest neighbors. Our study reveals the existence of a picosecond cooperative process in water, whose nature could be related to the cooperative rearrangements of several water molecules. This process may act as a precursor to large-scale transport related to viscosity. Our results help to improve the understanding of general transport mechanisms at the nanoscale, which can be useful for biomedical technologies or the development of nanofluidic devices.

## Graphical abstract

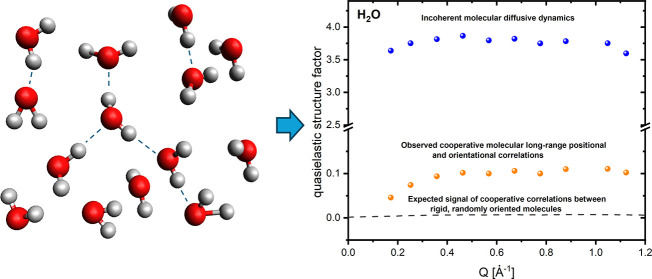

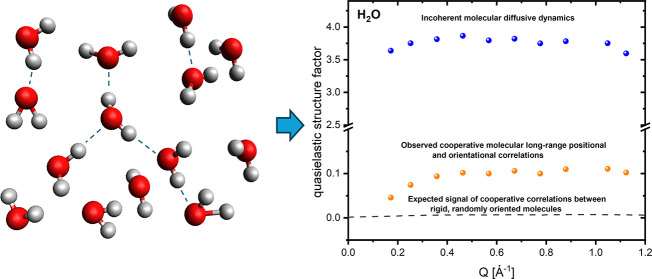

Water is one of the most abundant substances on earth and is an indispensable element for life on our planet, consisting of one oxygen atom and two hydrogen atoms arranged in a V-shape. Despite this apparent simplicity, water exhibits complex behavior, with approximately 75 known anomalous properties, setting water apart from other molecular liquids.^1^ It is widely believed that the nanoscale mechanisms behind most of water’s anomalous features can be traced to the existence and properties of hydrogen bonds (HBs), which arise from electrostatic and van der Waals intermolecular interactions^1^ between negatively charged oxygen atoms and positively charged hydrogen atoms of neighboring molecules. Each water molecule can form four hydrogen bonds with nearby molecules, ideally arranged in a tetrahedral configuration. These hydrogen bonds hold water molecules together, acting like a form of molecular “glue”, which, in turn, enhances the cooperative behavior of the liquid.

While the self-molecular dynamics of water has been well-studied, exploring its cooperative aspects remains challenging due to the absence of long-range order and the often short lifetimes of molecular correlations. Therefore, most investigations into nanoscale cooperativity were limited to structural effects.^2–5^ Theoretical studies^6–10^ have focused on cooperative dynamics, with molecular dynamics simulations of liquid water suggesting the formation of transient dynamical basins or cages formed by neighboring molecules,^11–15^ as well large collective rearrangements of water molecules, sometimes involving at least 9 water molecules.^8,16^ Recently, a two-state water model has been proposed, according to which water’s structure can be described as undergoing spontaneous fluctuations between regions with two distinct local structural arrangements^17^ or between regions with different degrees of cooperativity.^4,9^ In contrast, only a few remarkable examples of experimental work^18–23^ on cooperative dynamics in liquids were so far successful, leading to large gaps in understanding of cooperative aspects of water nanoscale structure and dynamics.

Neutron scattering is well-suited for investigation of dynamics phenomena at the nanoscale. The measured signal is directly related to the dynamic structure factor $S\left(\overset{⃗}{Q,}\omega \right)$ , that is a Fourier transformation of the Van Hove time-dependent pair correlation $G_{pair}\left(\mathrm{r⃗},t\right)$ and the time-dependent self-correlation $G_{self}\left(\mathrm{r⃗},t\right)$ functions.^24,25^ The pair-correlation function $G_{pair}\left(\mathrm{r⃗},t\right)$ describes cooperative molecular dynamics (see Supporting Information), while the self-correlation function $G_{self}\left(\mathrm{r⃗},t\right)$ follows the motion of the same particle. The Fourier transformations of $G_{pair}\left(\mathrm{r⃗},t\right)$ and $G_{self}\left(\mathrm{r⃗},t\right)$ give rise to so-called coherent and incoherent dynamic structure factors, respectively, whose contributions to the total measured signal, the double differential scattering cross section $\frac{d^{2}\sigma }{d\Omega dE}$ are weighted by corresponding scattering cross sections σ_*coh*_ and σ_*inc*_:

1 $$\frac{d^{2}\sigma }{d\Omega dE}=\frac{N}{4\pi }\frac{k_{sc}}{k_{inc}}\left[\sigma_{COH}S_{COH}\left(\mathrm{Q⃗},\omega \right)+\sigma_{INC}S_{INC}\left(\mathrm{Q⃗},\omega \right)\right]$$

The weight factors, namely, the coherent and incoherent scattering cross sections σ_*coh*_ and σ_*inc*_ are determined by the neutron scattering lengths *b*_sc_, which are element-specific and can vary significantly between different isotopes of the same chemical element (see Supporting Information 1). For D_2_O σ_*coh*_/molecule is about 10 times larger than σ_*inc*_/molecule making D_2_O mostly a coherent scatterer. In contrast, the incoherent scattering cross section of H_2_O is several orders of magnitude larger than its coherent scattering cross section.

Teixeira, Bellissent-Funel, and Chen were among the first to apply incoherent neutron spectroscopy to investigate water dynamics.^26^ Cooperative vibrational dynamics and sound waves were studied using neutrons and X-rays.^3,27^ Arbe and collaborators used D_2_O high coherent scattering cross section to investigate cooperative relaxation dynamics.^20,21^ However, investigations of H_2_O were hindered up to now by a very weak signal of a coherent component. Given the reported differences in hydrogen bond formation between D_2_O and H_2_O,^28–30^ exploring light water is crucial for a deeper understanding of cooperative dynamics. Here, we present the results of our investigation of the nanoscale cooperative molecular dynamics in light and heavy water using polarization analysis in neutron spectroscopy. This approach has, for the first time, enabled the unambiguous detection and successful separation of cooperative water dynamics from stochastic self-molecular motion in H_2_O, extending up to 10 ns.

Polarized neutron spectroscopy uses neutron spins, which are sensitive to the nuclear spins and contribute in this way to the scattering process.^24^ This feature can be exploited in nonmagnetic systems to separate coherent from incoherent nuclear scattering signals, which is precisely the approach taken in this study. The spins of incoming neutrons are aligned along the quantization axis by means of a device called a neutron polarizer and maintained via a magnetic guide field along the neutron trajectory.^24,31^ The neutron spin direction can be reversed by a device, known as a flipper. During the scattering process neutron–nuclear spin interactions with the sample can alter the neutron spin direction.^31^ In most common conditions, such as those considered in this work, the spins of the nuclei are randomly oriented without correlation to the spin direction of other nuclei; therefore, the spin-dependent scattering is of uncorrelated, i.e., incoherent character. The resulting probability for a neutron to reverse its spin direction due to the interaction with nuclear spin is 2/3.^31^ The spin-independent part of the neutron interaction with nuclei is of coherent character because it is the same for all nuclei of the same isotope.^24^ The polarization of a scattered neutron beam can then be analyzed using a polarization analyzer.^31^ In nonmagnetic, isotropic scattering systems like water, it is sufficient to measure the detected neutron intensity as a function of the flipper status: “switched on” when the neutron spins’ direction is reversed compared to their reference direction with respect to the guide field, and with the flipper “switched off” when there is no change of spin direction of the incoming neutrons. The polarization analyzer allows for the differentiation of neutrons scattered without change of spin direction (nonspin-flip, NSF) and those with spin direction reversed in the scattering process (spin-flip, SF).^27^ The measured signals can be presented as

2 $$S_{NSF}\left(Q,\omega \right)=S_{COH}\left(Q,\omega \right)+\frac{1}{3}S_{INCOH}\left(Q,\omega \right)$$

3 $$S_{SF}\left(Q,\omega \right)=\frac{2}{3}S_{INCOH}\left(Q,\omega \right)$$

These equations allow us to determine coherent and incoherent signals as

4 $$S_{COH}\left(Q,\omega \right)=S_{NSF}\left(Q,\omega \right)-\frac{1}{2}S_{SF}\left(Q,\omega \right)$$

5 $$S_{INC}\left(Q,\omega \right)=\frac{3}{2}S_{SF}\left(Q,\omega \right)$$

For our study we initially used the polarized neutron spectroscopy option implemented on the time-of-flight spectrometer NEAT in Berlin^32–37^ (the details are given in the Methods section and in Supporting Information). As a result, we could separate the coherent and incoherent signals in H_2_O (see Figure 1). However, the measured coherent dynamics, characterized by energy broadening, could not be reliably resolved due to the weak signal.

**Figure 1:**
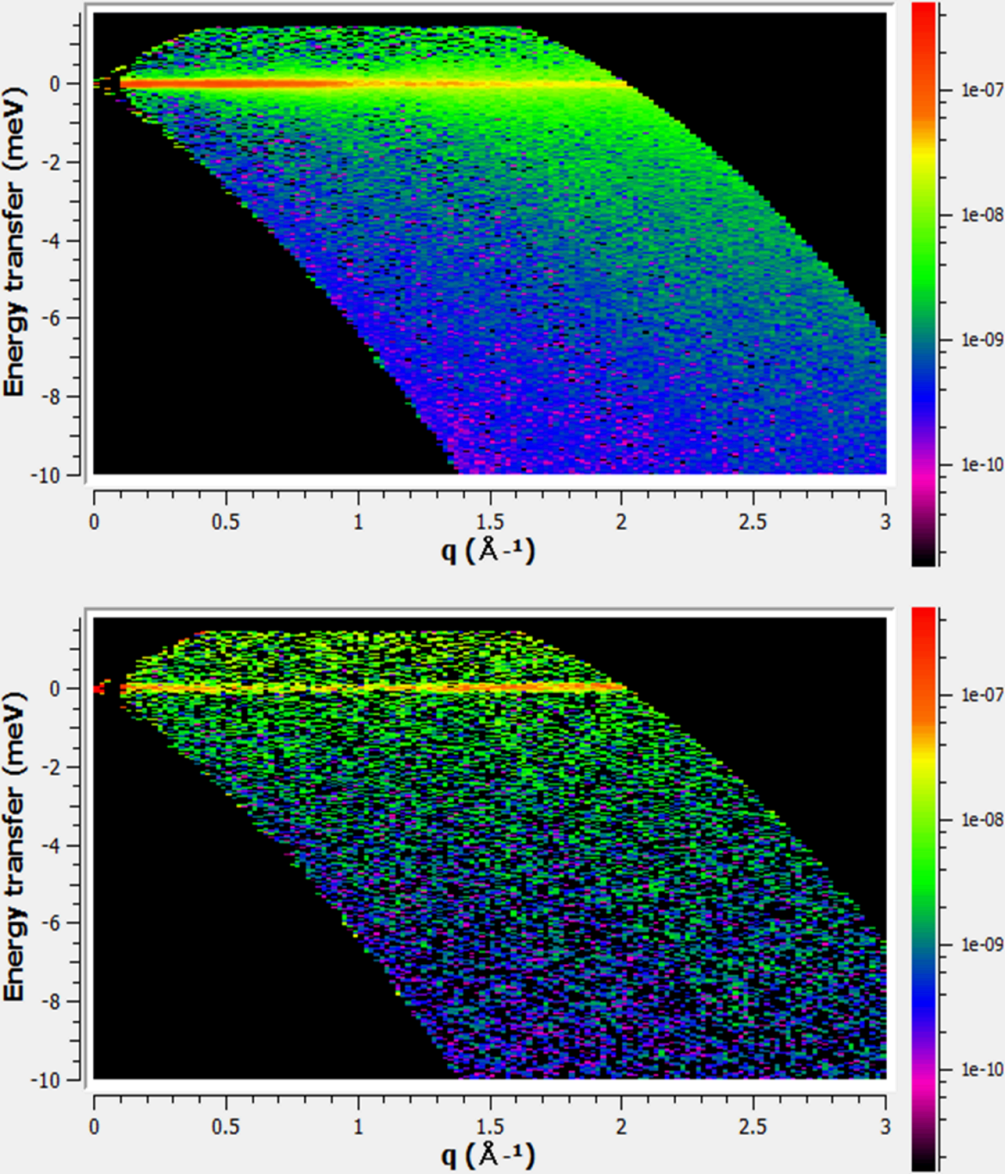
Incoherent *S*_*INC*_(*Q*,ω) (a) and coherent *S*_*COH*_(*Q*,ω) (b) structure factors, separated with the aid of polarization analysis on the neutron time-of-flight spectrometer NEAT. The differences between the two signals are clearly visible: the incoherent dynamics exhibit pronounced broadening, while the coherent signal is much weaker and evolves more slowly. The parameters extracted for the incoherent dynamics from the NEAT data were subsequently used in the next stage of the analysis, applied to data obtained through polarization analysis using neutron spin echo, as described in the main text.

Therefore, we developed a new approach for polarization analysis using neutron spin echo (NSE) spectroscopy.^38^ This approach allows us to measure the relaxation spectrum over a longer time scale up to >1 ns, compared to <0.1 ns accessible at NEAT. NSE works with the neutron spin,^39^ and the measured NSE signal, related to the intermediate scattering function *I*_*tot*_(*Q*,*t*), can be then presented as

6 Itot(Q,t)=SCOH(Q)ICOH(Q,t)−13SINC(Q)IINC(Q,t)SCOH(Q)−13SINC(Q)

where the intermediate scattering functions are normalized as *I*(*Q*,τ = 0) = 1, and *S*_*COH*_(*Q*) and *S*_*INC*_(*Q*) are absolute weight factors representing the contribution of coherent and incoherent signals, respectively. To determine *S*_*COH*_(*Q*) and *S*_*INC*_(*Q*), we used the WASP spectrometer^38^ and measured count rates of a fully polarized beam and a fully depolarized beam without any other change to its intensity or angular distribution using a specially established measurement procedure (details are given in the Methods and Supporting Information 3). As a result, we directly determined the total fractions of coherent and incoherent scattering with very high precision, integrated over the effective instrumental energy transfer window of WASP. The width of effective instrumental window Δ*Ε* was in our case of about ±2.5 meV.

For the analysis of the incoherent signal we have applied the model suggested previously^26^ and used since then very successfully in many QENS studies.^40–42^ The model assumes that a water molecule is undergoing a diffusion motion, realized through the jump-diffusion mechanism: the molecule resides at one place during the time τ_0_ before jumping to another position. In addition, water molecules rotate with characteristic time τ_*ROT*_, which is supposed to be related to the lifetime of hydrogen bonds. According to this model, the incoherent neutron scattering signal in the time domain *I*_*INC*_ *(Q,t)* is a product of the reorientational *y*_*ROT*_ *(Q,t)* and long-range translational diffusive *y*_*DIF*_ *(Q,t)* relaxation contributions, decoupled from each other. In this case the incoherent scattering function can be written as

7 $$I_{INC}\left(Q,t\right)=y_{ROT}\left(Q,t\right)\times y_{DIF}\left(Q,t\right)$$

8 $$y_{ROT}\left(Q,t\right)=A_{0}\left(Q\right)+A_{1}\left(Q\right)e^{-t/3\tau_{ROT}}+A_{2}\left(Q\right)e^{-t/\tau_{ROT}}$$

9 $$y_{DIF}\left(Q,t\right)=e^{-\Gamma \left(Q\right)t},with,\Gamma =\hbar \frac{D_{self}Q^{2}}{1+D_{self}Q^{2}\tau_{0}}$$

where *A*_*i*_(*Q*) stand for so-called elastic incoherent structure factors, given by spherical Bessel functions *j*_*i*_^2^(*b*) with the argument *b* = *Q* × *R*_*OH*_, where *R*_*OH*_ = 0.98 Å is the distance between the oxygen and hydrogen in the covalent bond:

10 $$A_{0}\left(Q\right)=j_{0}^{2}\left(b\right),A_{1}\left(Q\right)=3j_{1}^{2}\left(b\right),A_{2}\left(Q\right)=5j_{2}^{2}\left(b\right),A_{0}\left(Q\right)+A_{1}\left(Q\right)+A_{2}\left(Q\right)=1$$

The coherent intermediate scattering function can be well-described by a stretched exponential function, characterized by the relaxation time τ_COH_ of the relaxation process:

11 $$I_{COH}\left(Q,t\right)=e^{-{\left(\frac{t}{\tau_{COH}}\right)}^{\beta }}$$

The data sets obtained by polarization analysis on the time-of-flight spectrometer and on the NSE spectrometer were treated separately to avoid scaling effects, which often appear, when spectra from different instruments are combined. Therefore, the model described by eqs 7–([Disp-formula eq11]) has been converted to the *(Q,ω)* domain (Supporting Information 2).

As a first step, we applied the newly developed approach of polarization analysis using NSE to explore the D_2_O dynamics. Figure 2a shows coherent and incoherent structure factors *S*_*COH*_ *(Q)* and *S*_*INC*_ *(Q),* measured directly and independently of any model assumptions. The time-resolved coherent and incoherent signals in D_2_O contribute with opposite signs to the spectra, as shown in Figure 3. This leads to pronounced oscillations up to *Q* ∼ 1 Å^–1^, when the coherent signal is sufficiently large to surpass the incoherent signal. The fitting with eq 6 and with β = 1 provided good agreement with the data in the entire *Q* range, allowing us to calculate relaxation times for all three processes: τ_*ROT*_, τ_*DIF*_, τ_*COH*_ (Figure 5a). The obtained values are in excellent agreement with relaxation times established previously by Arbe^20,21^ and collaborators confirming the validity of our approach: τ_*ROT*_ is around 0.5 ps; the relaxation times for cooperative relaxation range mostly between 1 – 1.7 ps at 298 K.

**Figure 2:**
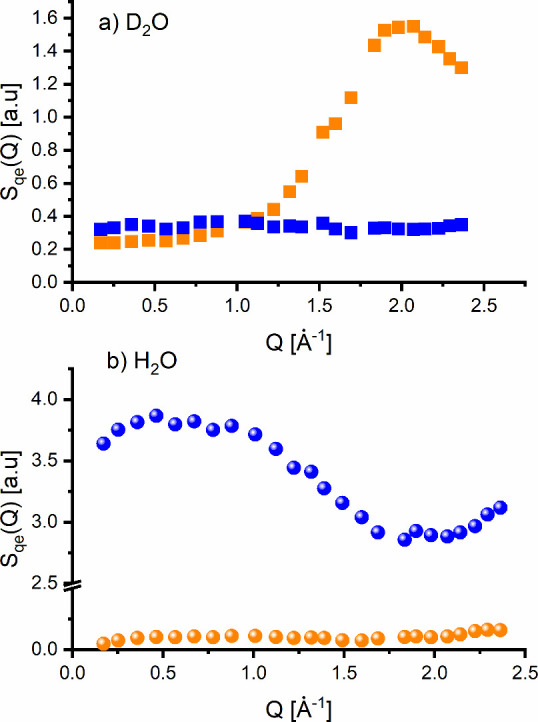
Coherent (orange symbols) and incoherent (blue symbols) quasielastic structure factors measured directly in D_2_O (a) and H_2_O (b) using polarization analysis combined with neutron spin echo (NSE) spectroscopy on the WASP spectrometer. The *Q*-dependence of the incoherent signal in D_2_O and H_2_O arises from the limited energy window accessible to the NSE spectrometer, which excludes contributions from molecular vibrations and phonon-like modes. In the intermediate *Q*-range of 0.5–1 Å^–1^, the observed coherent signal amounts to approximately 3–5% of the incoherent signal.

**Figure 3:**
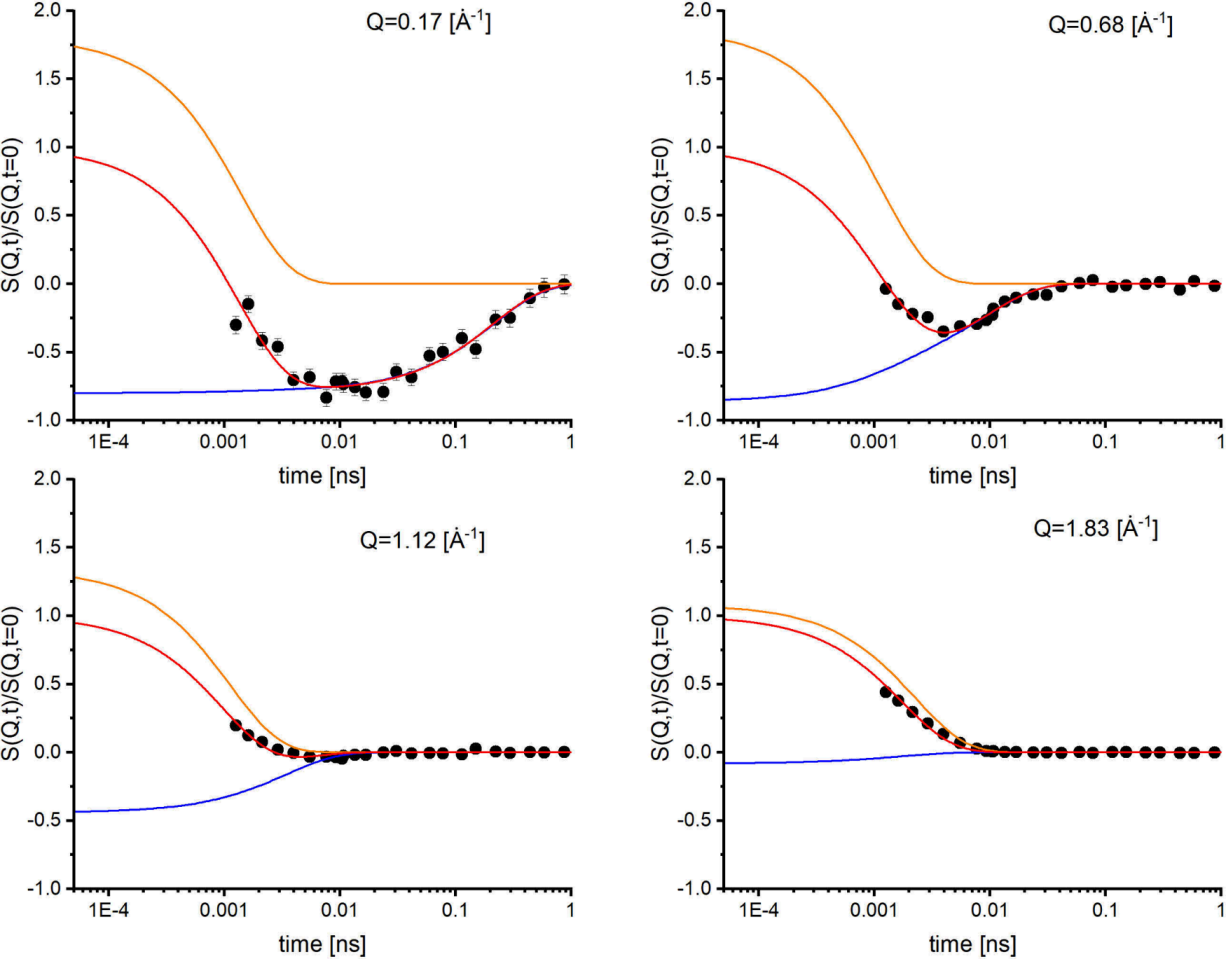
NSE spectra of D_2_O (black symbols) measured on WASP at selected *Q* values, along with fits (red line) using the model described by eqs 7–([Disp-formula eq11]) in the main text. The incoherent contribution (blue line) is predominantly negative, while the coherent contribution (orange line) is mostly positive, facilitating a clearer separation between the two components.

For the investigation of H_2_O water, we initially applied polarization analysis using time-of-flight spectroscopy on a NEAT spectrometer. This gave us the possibility to measure high-frequency dynamics up to 20 meV in addition to the quasielastic range. As a result, we could separate the incoherent and coherent signals and verified the contribution of the vibrational excitations, i.e., sound waves. Previous investigations revealed two sound wave branches.^27^ The coherent signal obtained on NEAT shows no detectable contribution of these vibrations to the quasielastic energy range Δ*Ε* = ±2.5 meV in light water. For the analysis of the quasielastic incoherent dynamics, we applied the model described before by eqs 7–([Disp-formula eq11]) and converted it into the energy domain. The average reorientational time ⟨τ_*ROT*_⟩ ∼ 1 ps calculated from the fit at 298 K agrees well with the literature data.^26^ Lowering the temperature to *T* = 285 K resulted in slightly larger rotational ⟨τ_*ROT*_⟩ ∼ 1.38 ps (Figure S5).

Next, we applied polarization analysis on the NSE and detected a clear coherent signal in H_2_O in the entire *Q*-range. This marks the first direct measurement of the inelastic coherent signal in H_2_O in the quasielastic range of about ±2.5 meV (Figure 2b). We verified potential spurious scattering contributions to the spectra, particularly, double incoherent scattering. Incoherent scattering primarily contributes to spin-flip scattering, while coherent scattering leads to nonspin-flip processes. In the case of double scattering, the two spin-flip events would appear as nonspin-flip scattering, potentially resembling coherent signals in polarization analysis. The degree of polarization of the double incoherent scattering is (−1/3)^2^ = 1/9; i.e., 8/9 fraction of the beam appears as unpolarized and 1/9 as polarized in the NSF direction. The multiple scattering contribution varies with the sample’s scattering strength and can be influenced, for example, by changes in sample thickness. Because incoherent scattering is generally independent of *Q*, the multiple scattering contribution can be assumed to remain constant across the measured *Q* range. Hence, to directly estimate this contribution experimentally, we measured samples with two different thicknesses (0.1 and 0.05 mm) and at an incoming neutron wavelength of 6 Å. The measured intensities were normalized to the total incoherent scattering of the samples to achieve reliable absolute calibration of the spectra, which was affected little by multiple scattering. The multiple scattering was identified by the deviation of the scattering signal intensity from proportionality with the mass of the sample. The results show that for the samples with 0.1 mm thickness the multiple scattering contributes to the measured apparent coherent quasielastic structure factor by a *Q* independent 3.15 ± 0.07% fraction of the incoherent single scattering structure factor. Note that since the WASP polarization analyzer does not transmit neutrons with energy more than a certain cutoff energy, the total scattering of the H_2_O sample can be significantly higher at room temperature with a substantial part of the inelastic scattering extending even beyond the range of 30 meV scattered neutron energy. To analyze the time structure of the multiple scattering contribution, we compared measured NSE spectra of two different sample thicknesses (and hence different multiple scattering contributions) and observed no difference in the line shape of all spectra (Figure S4). This suggests a strong increase in the inelasticity of double incoherent scattering compared to single incoherent scattering, leading to faster dynamics and a broader apparent quasielastic line width. The final change of neutron energy of the double scattering can however still fit into a quasielastic analyzer transmission window and contribute to quasielastic structure factors, even if the energy changes for two individual scattering processes could be much higher. Thus, the contribution of the doubly incoherent scattering to our measured NSE signal can be considered as zero in the NSE time range accessible in our experiment >0.001 ps, i.e., energies < ∼0.6 meV. The measured spectra were corrected for the determined contribution of the multiple scattering. Corrected coherent structure factors (Figure S4 in Supporting Information 4) show good agreement between each other, with independence from the sample thickness and the incoming neutron wavelength, confirming the validity of our approach.

The corrected coherent and incoherent structure factors of H_2_O integrated over energy range of Δ*Ε* = ±2.5 meV are shown in Figure 2. The incoherent dynamics in H_2_O and D_2_O water does not show large differences and is dominated by self-diffusion contribution, showing a slight decrease with increasing momentum transfer. This is the result of the integration in the limited energy and momentum transfer ranges. The total incoherent scattering, when integrated in the infinite energy range and in the solid angle of 4π, is independent of *Q*. However, in our experimental window of Δ*Ε*= ±2.5 meV molecular vibrations and phonon-like motion, whose intensities are proportional to *Q* ^2^, were outside the studied energy range, which lead to the slight *Q* dependence of the quasielastic structure factors. Coherent quasielastic structure factors show clear differences in D_2_O and H_2_O originating from a difference in neutron scattering lengths in water isotopes. The scattering cross-section σ of an element is related to scattering length *b*. For a molecule in a liquid at distances larger than the typical intramolecular bond lengths the coherent molecular cross section can be approximated as $\sigma_{coh,mol}=4\pi {\left(\underset{j}{\sum }{\mathrm{b̅}}_{j}\right)}^{2}$ where the index *j* runs over the atoms within one individual molecule (see Supporting Information 1).

For the calculation of the coherent scattering cross section, it is important to consider the sign of the scattering length, as coherent scattering arises from the interference of waves scattered by all nuclei: they add up constructively if the scattering lengths have the same sign and destructively if they have opposite signs. In D_2_O the scattering length of deuterium atoms is *b*_*coh*_^*D*^ = 6.671 fm, and of oxygen *b*_*coh*_^*O*^ = 5.803 fm. This leads to the pronounced coherent signal and a large peak at *Q* ∼ 1.8–2 Å ^–1^ as a mark sign of a well-developed short-range order. In H_2_O the coherent signal is flat in almost the entire *Q* range from 0.2 to 2.4 Å^–1^ resulting from differences in sign of the neutron scattering length *b*_*coh*_ for H and O. The coherent scattering length for H is negative *b*_*coh*_^*H*^ = −3.74 fm, while for O it is positive *b*_*coh*_^*O*^ = 5.803 fm. Since the short-range originates from strong atomic correlations, mainly governed by the correlations between the centers of gravity of randomly oriented molecules, the contributions of H and O atoms effectively cancel each other and no peak in H_2_O is observed in the coherent scattering in the range of *Q* < 2.2 Å^–1^.

Remarkable is, however, that despite of the peak absence at *Q* = 1.8–2 Å^–1^ the coherent signal in H_2_O in the entire *Q* range is much higher as one would expect for the case of rigid, non-interacting and randomly oriented molecules. While for the latter the coherent signal (σ_*coh*,*mol*_ = 0.35 barn) would theoretically be only about ∼0.2% of the incoherent signal (σ_*incoh*,*mol*_ = 160.52 barn), the observed coherent signal is about 2.5–3% of the incoherent signal. These differences are clearly pronounced at the length scale of the intermediate *Q* range order (<1–1.2 Å^–1^). Considering that intermediate range order corresponds to distances comparable up to several times the average molecular distances, our observation is the clear evidence *that the intermolecular correlations in water are not just the short-range, local correlations between atoms within one or two H*_2_ *O molecule, but they also spread to neighboring and more distant molecules, leading to cooperative structural and dynamical fluctuations.* Due to the high scattering cross section of the proton, even slight differences in the motions of hydrogen and oxygen atoms, caused by long-range and orientational correlations, could lead to a higher coherent neutron scattering signal. The proposed spontaneous bond swapping within molecular groups^9,43^ and the sharing of a H atom in a hydrogen bond between two molecules are just maybe the best-known eminent examples of this kind of richer, wider atomic correlation, as opposed to the rigid molecule picture, where the atomic correlations are mainly governed just by the correlations between the centers of gravity of molecules. The enhancement of the signal can also be caused by correlations between preferential orientations of several molecules and rearrangements of hydrogen bonds. The existence of such interactions, ranging from directed hydrogen bonds to dipole–dipole interactions and to quantum nuclear effects, and the formation of molecular ordering at distances going beyond the second coordination shell were also suggested by several theoretical studies.^3,44^ The observation of enhanced coherent signal in H_2_O is the major difference to D_2_O where the signal follows a more classical hydrodynamic approach.^45^ This can be explained by reported differences^2,28^ in molecular bonds of H_2_O and D_2_O: While D_2_O is a more symmetrical molecule, OH molecular bonds in H_2_O are slightly asymmetrical making bond sharing, quantum effects,^46^ and cooperative interactions more pronounced in neutron scattering spectra.

For the analysis of the time-dependent H_2_O data (Figure 4) obtained by NSE we used the values of relaxation time τ_*ROT*_ of reorientational motion and the relaxation time τ_*TRANS*_ for diffusive motion established before for H_2_O in our NEAT experiment for both temperatures studied, 298 and 285 K. Since *S*_*COH*_(*Q*) and *S*_*INC*_(*Q*) were directly measured and elastic incoherent structure factors *A*_*i*_( *Q* ) were calculated, the fit mostly uses only one free fitting parameter τ_*COH*_, which increased substantially the fit quality. The determined relaxation times are presented in Figure 5b and Figure S5. The value of activation energy of the cooperative process of ∼8 kcal/mol (see Figure S7) is higher than for the singular hydrogen bond pair, which supports the hypothesis of cooperative behavior.

**Figure 4:**
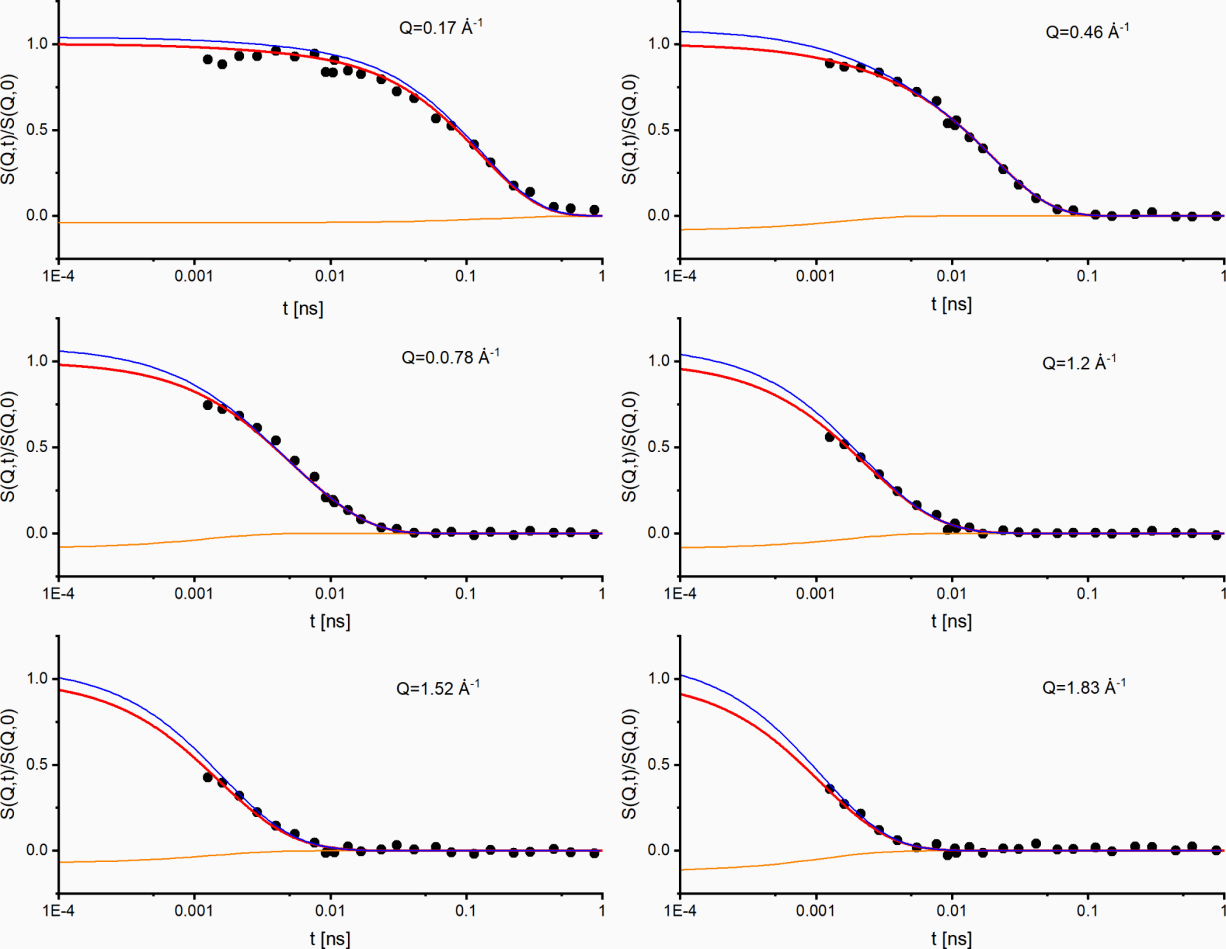
NSE spectra of H_2_O (black symbols) at 298 K measured on WASP at selected *Q* values. The red line shows the full fit using the model described by eqs 7–([Disp-formula eq11]) in the main text. The corresponding incoherent (blue line) and coherent (orange line) contributions are also shown.

**Figure 5:**
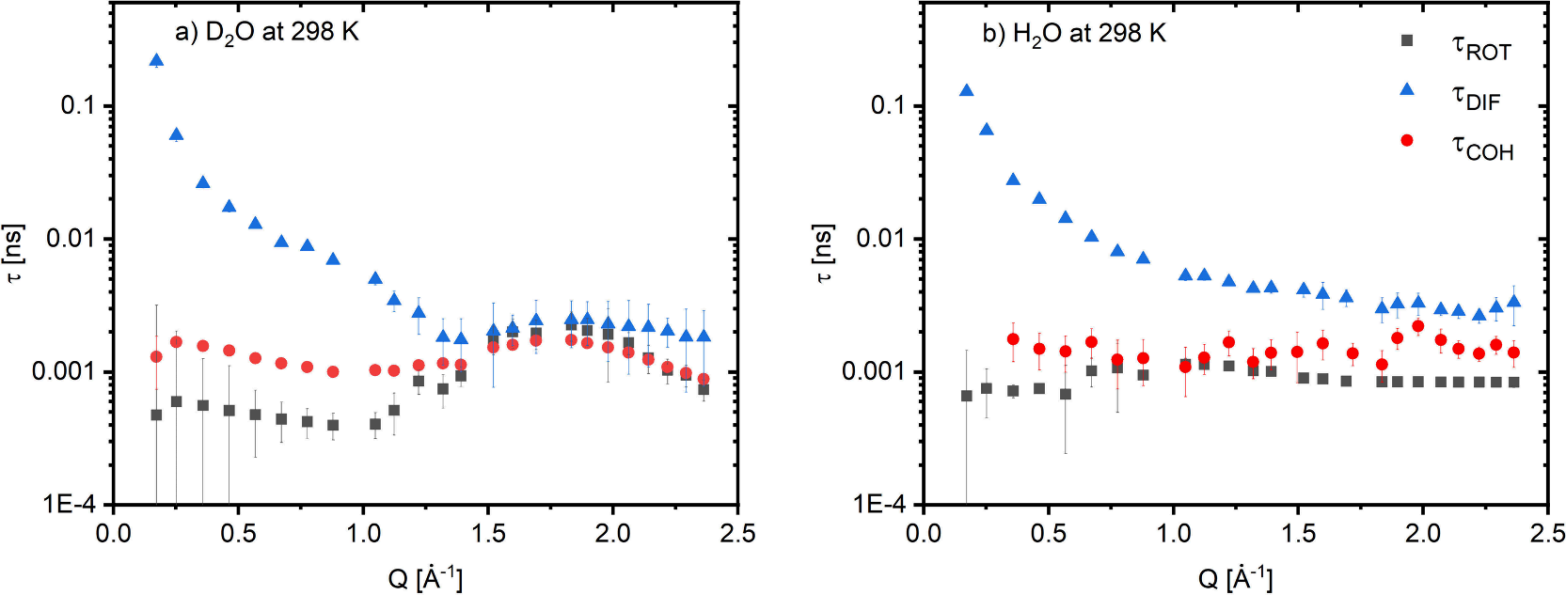
Experimentally determined relaxation times for reorientational τ_*ROT*_, coherent τ_*COH*_, and translational, self-diffusion τ_*DIF*_ processes in D_2_O (a) and H_2_O (b) at 298 K obtained using polarization analysis combined with the neutron spin echo technique. Coherent relaxation times are systematically longer than incoherent reorientational τ_*ROT*_, reflecting the collective nature of the underlying motions.

What could be the possible physical nature of the observed cooperative dynamics? Confinement can enhance cooperativity and make cooperative processes more visible, even in incoherently scattering systems. Indeed, for water confined into sub-nanometer linear pores we see the formation of ordered water chains with distances between nearest neighbors of 3.4–4 Å, which are substantially longer than the regular hydrogen bonds in bulk water. This ordered and less dense water shows a high degree of cooperativity in the molecular nanoscale dynamics, displaying fast cooperative rearrangements between nearest neighbors as a first step of a large-scale diffusive transport.^47,48^ In the course of such a rearrangement, molecular positions are changed, the hydrogen bonds are broken and formed again, and water molecules are reoriented in the new environment. Note that fast cooperative local rearrangements of ionic groups were also reported in the glass forming liquids as a precursor to the large-scale viscosity-related molecular flow.^22^ Thus, one can speculate that also the cooperative signal, reported here, could be related to similar spontaneous cooperative molecular rearrangements of several water molecules.

Molecular dynamic simulation studies estimate the characteristic time of local relaxion within dynamic cooperative basins as 1 ps at room temperature.^11,43^ This value agrees well with the characteristic relaxation times of the coherent signal found here, which range between 1.2–2 ps (Figure 5a,b) at 300 K and 2–3 ps at 285 K (Figure S5). The *Q* dependence of the signal shows two distinctive regions hinting at the existence of two different mechanisms. Thus, for *Q* > 1 Å^–1^ the *Q* dependence of relaxation times can be described as τ_coh_(*Q*) ∝ const × S_coh_(*Q*). At *Q* < 1 Å^–1^ the relaxation times slightly increase with decreasing *Q*’s, which is the characteristic of translational diffusive motion. Using the relation *Q* = 2π/*l* one could approximate the length *l* ∼ 6 Å where these changes take place. A similar value and two different regimes in cooperative molecular ordering were found in the recent molecular dynamic simulation study.^44^

To conclude, we investigated the cooperative dynamics of water using neutron polarization analysis in the time domain, extending the time domain up to 10 ns. A major challenge in studying these dynamics is the need to disentangle molecular self-motion, which dominates incoherent neutron scattering, from cooperative dynamics, which manifest in the coherent neutron scattering signal. Until now, this distinction has been experimentally hindered by the inherently weak coherent signal in H_2_O.

By employing advanced quasielastic neutron scattering techniques and developing a novel approach to polarization analysis using neutron spin echo, we achieved unprecedented sensitivity in detecting and analyzing the coherent neutron scattering signal in light water. Our results reveal that the coherent quasielastic signal is an order of magnitude stronger than expected for rigid, randomly oriented molecules at the length scale of intermediate-range order. This provides clear evidence that intermolecular interactions in water extend beyond simple short-range correlations between the centers of mass of individual molecules and instead involve complex, long-range cooperative effects. Beyond self-diffusion and molecular rotation, we identified a picosecond cooperative process in water, likely corresponding to the collective rearrangement of multiple neighboring water molecules, including hydrogen bond network reorganization. This process may act as a precursor to large-scale molecular transport and viscosity-related dynamics. Our results offer new insights into the general transport mechanisms and nanoscale dynamics in water. Moreover, the observed cooperative dynamics could influence the formation of intermolecular bonds with the environment, with potential applications in the development of new biomedical technologies and nanofluidic devices.^49,50^ Additionally, the newly developed approach for polarization analysis using neutron spin echo opens exciting possibilities for directly measuring coherent and incoherent signals in systems with mixed scattering.

## Experimental Methods

*Samples*. We used ultrapure hydrogenated water and deuterated water produced by Aldrich Chemical Co. Inc. with a deuterium content of 99.9%. The samples were placed in aluminum sample holders in hollow cylinder geometry with thicknesses adapted for H_2_O and D_2_O to avoid multiple scattering effects. The sample thickness for H_2_O was 0.1 mm and 0.05 mm, and for D_2_O it was 0.4 mm. The samples in both experiments were measured at 298, 285, and 268 K.

*Polarization analysis using neutron TOF and NSE spectroscopies*. For polarization analysis using spectrometer NEAT we used the specially developed experimental setup.^34,35^ The analysis of the polarization in the scattered neutron beam has been realized using a donut-shaped glass cell with a wedge-shaped cut-out to pass the incident neutron beam.^36,37^ Further details are given in Supporting Information 2. For the implementation of polarization analysis using neutron spin echo we used the NSE spectrometer WASP^38^ at Institute Laue-Langevin in Grenoble, France. Its experimental setup allows determination of the spin flip (SF) and spin conserving (nonspin flip, NSF) components of the scattered beam. More details are given in Supporting Information 3.

## Supplementary Material



## Notes

Details on polarization analysis using time-of-flight and neutron scattering techniques and data treatment (PDF)
