# Supplementary material for: Structural Fluctuations at Nanoscale and Cooperative Molecular Dynamics in Bulk Water
Source: J Phys Chem Lett. 2025 Jun 4;16(23):5835–43. doi: 10.1021/acs.jpclett.5c00735 (PMC12169651; doi:10.1021/acs.jpclett.5c00735)
Supplement: Supplementary file 1 [file jz5c00735_si_001.pdf]

Supplemental materials

Structural fluctuations at nanoscale and cooperative molecular dynamics in bulk water

Margarita Russina[1], Gerrit Günther[1], Bela Farago[2], Earl Babcock[3], Zahir Salhi[3], Alexander Ioffe[3] and Ferenc Mezei[4]

[1] *Helmholtz Zentrum Berlin, Hahn-Meitner Platz 1, 14109 Berlin, Germany*

[2] *Institute Laue-Langevin - 71 avenue des Martyrs, CS 20156, 38042 GRENOBLE Cedex 9 - France*

[3] *Jülich Centre for Neutron Science (JCNS) at Heinz Maier-Leibnitz Zentrum (MLZ), Forschungszentrum Julich GmbH, 85747 Garching, Germany*

[4] KFKI Campus, H-1525 Budapest, Konkoly Thege u 29-33, Hungary

Email: margarita.russina@helmholtz-berlin.de

1. Coherent and incoherent neutron scattering from liquids

In the scattering event of a neutron by a nucleus the incident neutrons can be described as plane waves e^{ikr}. Interactions with nucleus leads to the generation of spherical waves $\frac{b}{r}e^{ik'r}$. The amplitude of the scattered spherical wave b/r *depends* on the strength of the interactions between the neutrons and nucleus given by the scattering length b. In general, the scattering length can be of positive and negative sign, originating from repulsive and attractive interactions correspondingly. The final scattering pattern is the interference of all scattered waves, which can be written for monoatomic liquid, using the angular brackets for the ensemble average over all possible configuration of the nuclei in the sample as:

$$I(\vec{Q},\omega) = \frac{1}{2\pi\hbar}\frac{k_{sc}}{k_{inc}}\sum_{i,j} b_i b_j \int_{-\infty}^{\infty} \langle e^{-i\vec{Q}\,\vec{r}_i(0)}\, e^{-i\vec{Q}\,\vec{r}_j(t)} \rangle\, e^{-i\omega t}\, dt \qquad \text{(s.1)}$$

The term in brackets means the probability to be scattered by the nucleus *i* at the position r_i at the time zero and the nucleus j at the position r_j at the time t. In the equation the scattering length b can vary for different atoms, isotopes or due the presence of nuclear spin. For monoatomic liquid summing up over the total sample volume in the equation above we can write using $A_{i,j}$ as a notification of the integral in eq (s.1):

$$\sum_{i,j} \overline{b_i b_j}\, A_{i,j} = \sum_{i,j} \bar{b}^2\, A_{i,j} + \sum_j \left(\overline{b^2} - \bar{b}^2\right) A_{j,j} \qquad \text{(s.2)}$$

The first term is the contribution from scattered neutron waves from different pairs of nuclei or the same nuclei at different times which interfere with each other. It gives rise to so-called coherent scattering $\sigma_{coh} = 4\pi \sum_j \bar{b}_j^{\,2}$. The second term describes the waves scattered without interference with another nucleus, usually originating from differences between the nuclei randomly occupying equivalent positions in the sample structure. It is noted as incoherent scattering $\sigma_{inc} = 4\pi \sum_j \left(\overline{b^2} - \bar{b}^2\right)$.

The scattering low can be now presented as

$$I(Q,\omega) = I_{COH}(Q,\omega) + I_{INC}(Q,\omega) \qquad \text{(s.3)}$$

The scattering function can be derived from the time dependent correlation function Van Hove has introduced, which represent the probability to find a particle at the origin of coordinate system at time zero and at the position r at time t:

$$G(\vec{r},t) = \frac{1}{N}\sum_{i,j} \delta\left(\vec{r} - \left[\vec{r_i}(0) - \overrightarrow{r_j(t)}\right]\right) \qquad \text{(s.4)}$$

The time dependent correlation function can be split into pair-correlation function $G_{pair}(\vec{r},t)$ and self-correlation function $G_{self}(\vec{r},t)$. The pair-correlation function $G_{pair}(\vec{r},t)$ describes cooperative dynamics by giving a probability of finding a particle j at a distance $\vec{r}$ at time t knowing that a particle i is in the vicinity of the origin at time t=0, and describes therefore the cooperative microscopic dynamics. The $G_{self}(\vec{r},t)$ describes the probability

to find the same particle at various times and different positions, therefore follows the self-motion of one particle.

Van Hove has also shown that the dynamic structure factor $S(\vec{Q},\omega)$ can be presented as a Fourier transform of the corresponding time correlation function in space and time. Thus, we have:

$$S_{COH}(\vec{Q},\omega) = \frac{1}{2\pi\hbar}\int G_{PAIR}(\vec{r},t)e^{i(\vec{Q}\vec{r}-\omega t)}d\vec{r}dt \qquad \text{(s.5)}$$

and

$$S_{INC}(\vec{Q},\omega) = \frac{1}{2\pi\hbar}\int G_{SELF}(\vec{r},t)e^{i(\vec{Q}\vec{r}-\omega t)}d\vec{r}dt \qquad \text{(s.6)}$$

In most of the experiments the double differential scattering cross section is measured as a function of momentum transfer and energy, where the coherent and incoherent dynamic structure factor contributions are weighted by the coherent and incoherent scattering cross sections:

$$\frac{d^2\sigma}{d\Omega dE} = \frac{N}{4\pi}\frac{k_{sc}}{k_{inc}}\left[\sigma_{COH}S_{COH}(\vec{Q},\omega) + \sigma_{INC}S_{INC}(\vec{Q},\omega)\right] \qquad \text{(s.7)}$$

In neutron spin echo spectroscopy, the intermediate scattering function, which is the Fourier transform of time dependent Van Hove correlation function in space, is measured directly (in excellent approximation if the relative energy change of the neutron in the scattering process is small, typically < 10 %)

$$I(\vec{Q},t) = \int G(\vec{r},t)\, e^{i\vec{Q}\vec{r}}d\vec{r} = \hbar\int S(\vec{Q},\omega)e^{i\omega t}d\omega \qquad \text{(s.8)}$$

In non-monoatomic liquids, both coherent and incoherent scattering must be calculated as sums of contributions of partial dynamic structure factors, originating from all correlating atoms and molecules, each weighted by the corresponding scattering lengths. However, in a liquid that can be described as an ensemble of rigid, randomly oriented, and randomly positioned molecules, and at distances larger than the typical intramolecular bond lengths, the terms in the brackets ⟨ ⟩ in eq. s.2 approach 1 for atoms within the same molecule. In this case the coherent scattering $\sum_{jj'}\overline{b_{j'}b_j}$ becomes $(\sum_j\overline{b_j})^2$, with the index *j* running over the atoms within one molecule. With randomness in position and orientation of the molecules, all terms with *j* and *j'* belonging to different molecules average to zero. i.e. $(\sum_j\overline{b_j})^2$ gives indeed the coherent scattering cross section per molecule in an assembly of uncorrelated identical molecules.

For more details on the neutron scattering please see [1, 2]

2. Experimental details for polarization analysis using time- of -flight spectrometer NEAT

In our study we used the experimental setup for polarization spectroscopy installed on time-of-flight spectrometer NEAT. The polarization of incoming neutrons was realized by a polarizer surrounded by permanent magnets, a neutron spin flipper and a series of permanent magnets and solenoids to provide a magnetic guide field from polarizer to the sample[3, 4]. The polarizer itself consists of a V-shaped arrangement of neutron polarizing mirrors, which deflect neutrons with unwanted spin direction and let neutrons with desirable spin direction to continue their propagation inside the neutron guide. The spins of incoming neutrons were aligned horizontally in the direction perpendicular to the neutron propagation.

The analysis of the polarization in the scattered neutron beam has been realized using a donut shaped glass cell with a wedge-shaped cut-out to pass the incident neutron beam. The cell is filled with 1.5 bar of polarized ^{3}He gas[5, 6, 7] . The 3He-cell works like a filter: neutrons whose spin is antiparallel to those of ^{3}He atoms are preferentially absorbed. For the polarization of the ^{3}He we have built a setup at Helmholtz Zentrum Berlin, where a 70-80% polarization of ^{3}He-atoms in the cell has been achieved on a 24 hour time scale. On NEAT the neutron analyzer is surrounded by a coil system to create a magnetic guide field which maintains the polarization of the ^{3}He atoms and prevents depolarization of the passing scattered neutrons. The donut-like shape of the ^{3}He cell allowed for the analysis of the polarized neutrons over the entire large scattered angular range of the NEAT detector system simultaneously.

The polarization efficiency of the entire system is a product of the polarization efficiency of its components. The polarization and transmission of the neutron polarizer is constant in time. In contrast, the transmission of the ^{3}He cell is time and spin-direction dependent and can be described as:

$$T_{\uparrow} = A_0 e^{-6N_0\left(\frac{1}{2}+\frac{P_0}{2}e^{-ct}\right)} \qquad \text{(s.9)}$$

$$T_{\downarrow} = A_0 e^{-6N_0\left(\frac{1}{2}-\frac{P_0}{2}e^{-ct}\right)} \qquad \text{(s.10)}$$

Here A_0 is a normalization parameter, N_0 is the number of ^{3}He atoms along the neutron path in the cell, P_0 denotes the initial polarization of the ^{3}He atoms in the cell, c is the ^{3}He polarization time decay constant and t is the actual experimental time.

As mentioned before, in the experiment the intensity I of the scattered neutrons has been measured with the spin flipper switched on and off. In this case the transmission of the entire system C can be given as:

$$C_{OFF} = I_{\uparrow}T_{\uparrow} + I_{\downarrow}T_{\downarrow} \quad \text{(s.11)}$$

$$C_{ON} = I_{\downarrow}T_{\uparrow} + I_{\uparrow}T_{\downarrow} \quad \text{(s.12)}$$

The values were measured in the experiment directly using the neutron beam monitor for the transmitted beam behind the sample. Very important parameter is the flipping ratio R which is defined as

$$R = C_{ON}/C_{OFF} \quad \text{(s.13)}$$

Since in the polarized ^{3}He cell we used $T_{\uparrow} \ll T_{\downarrow}$, we can approximate R to be:

$$R = I_{\uparrow}/I_{\downarrow} \quad \text{(s.14)}$$

In the experiment the scattered intensity has been recorded for several hours, alternately for 30 min with the flipper switched on and off. Afterwards, data were grouped, normalized to the incoming neutron flux, detector efficiency and converted to momentum and energy transfer (Q,ω).

$S_{INC}(Q,\omega)$ and $S_{COH}(Q,\omega)$ scattering contributions were calculated from the measured $S_{ON}(Q,\omega)$ and $S_{OFF}(Q,\omega)$ signals as follows:

$$S_{INC}(Q,\omega) = \left[S_{OFF}(Q,\omega) - \frac{1}{R}S_{ON}(Q,\omega)\right] * \frac{3/2}{1-\frac{1}{2R^2}} \quad \text{(s.15)}$$

$$S_{COH}(Q,\omega) = \left[S_{ON}(Q,\omega) - \frac{1}{2}S_{OFF}(Q,\omega) - \frac{1}{2R}S_{INC}(Q,\omega)\right] * \frac{1}{1-\frac{1}{2R}} \quad \text{(s.16)}$$

The special challenge in this case comes from the use of polarized ^{3}He analyzer, whose performance changes with time as the polarization of ^{3}He nuclei decays with a time constant comparable to a day, while all the rest of the equipment works time independently. The critical issue here is how to compare spectra taken at different times.

With more than several orders of magnitude difference between $T_{\downarrow}$ and $T_{\uparrow}$ all the time, the polarizing gas filter always remained with nearly perfect polarization efficiency, but the transmission changed with time, namely a dropped by about factor of 5 in 48 hours for the preferentially transmitted neutron spin polarization. This has been monitored in the direct beam downstream from the sample and the analyzer with the transmission monitor. The normalization of all beam intensities coming from the sample and unavoidably crossing the analyzer ^{3}He gas container to a selected point in time (e.g., the time of the first scan after installation of the freshly polarized ^{3}He filter analyzer on the instrument) was in principle straightforward in view of the constantly monitored C_{ON} and C_{OFF} transmission counts, with one exception: reliable correction for the background.

The neutrons that cause the background can go through the analyzer (e.g. scattering from the sample holder) in which case the time dependence is the same as measured by the downstream beam monitor of the direct beam going through the polarization analyzer

gas. Another part of the background comes from neutrons from the environment and neutrons scattered around in the sample chamber, without going through the absorbing polarized ^{3}He analyzer. For this reason, the time dependence of the background needs to be split to a fixed part and a time dependent part. The experimental determination of the time dependence of the background required some fraction of the available beamtime for the experimental scans within the time of a period covered by one freshly polarized analyzer on the instrument. It proved to provide at some part of the detector area a major factor in the error of the corrected spectra for data analysis. The otherwise conventional correction of the measured sample spectra $S_{ON}(Q,\omega)$ and $S_{OFF}(Q,\omega)$ to the background, normalized with its time dependence to the reference time of the sample spectra, was performed before the so corrected $S_{ON}(Q,\omega)$ and $S_{OFF}(Q,\omega)$ were converted to $S_{INC}(Q,\omega)$ and $S_{COH}(Q,\omega)$, following eqs. (s.15) and (s.16) above.

The established procedure has been verified using the water sample confined into zeolite where the incoherent signal consisted predominantly of the water signal and the coherent part come from zeolite structure. In the case of the bulk water we have seen that this procedure has worked efficiently well in the majority of the scattering angles, with the exception of high scattering angles where the incoherent scattering amplitude become very low. In this case we have seen the appearance of an additional small elastic signal which was removed from the spectra by fitting.

In the experiment we used incoming neutrons with the wavelength of 6 Å, which resulted in the FWHM of the elastic resolution function of 100-110 μeV. For the primary data treatment we have used software package MANTID[8], which has been modified to incorporate polarization analysis routines developed by us. The collected data were grouped, normalized to the incoming neutron flux and corrected for the time dependent polarization and detector efficiency. Afterwards the background contribution has been removed and the data converted to momentum and energy transfer (Q,ω). In the final step coherent and coherent structure factors have been calculated.

For the data analysis we have used the model suggested before and described in the main text[9]. The model has been converted to the energy domain:

$$S_{INC}(Q,\omega) = y_{ROT}(Q,\omega) \otimes y_{DIF}(Q,\omega) \otimes y_{RES}(Q,\omega) \qquad \text{(s.17)}$$

$$y_{ROT}(Q,\omega) = A_0(Q) + A_1(Q)L_1\left(Q,\frac{\hbar}{3\tau_{ROT}}\right) + A_2(Q)L_2\left(Q,\frac{\hbar}{\tau_{ROT}}\right) \qquad \text{(s.18)}$$

$$y_{DIF}(Q,\omega) = L_0(Q,\Gamma) \quad L_i(Q,\Gamma) = \frac{2}{\pi}\frac{\Gamma}{4(x-x_c)^2+\Gamma^2} \quad \text{with } \Gamma = \hbar\frac{D_{self}Q^2}{1+D_{self}Q^2\tau_0} \qquad \text{(s.19)}$$

$$A_0(Q) = j_0^2(b) \quad A_1(Q) = 3j_1^2(b) \quad A_2(Q) = 5\,j_2^2(b) \quad A_0(Q) + A_1(Q) + A_2(Q) = 1 \quad \text{(s.20)}$$

The Figure S1 shows the experimental data obtained on NEAT and the fit using this model. From the Q-dependence of the quasielastic broadening Γ for the diffusive contribution $y_{DIF}(Q,\omega)$ we calculated the diffusion coefficient D_{self} and the residence time τ_0. From the fit we have: D_{self} =(1.69±0.07)x10^{-9} m^2/s and τ_0=1.94±0.0035 ps at 285K and D_{self} =(2.23±0.024)x10^{-9} m^2/s and τ_0=1.70 ±0.002 ps at T=298 K. The results, shown on the Figure S2 agree well with the literature data[9]. The relaxation times for rotational diffusion at 298 K were used in the fit. Considering that the Q-dependence of the rotational relaxation times at 298 K is relatively flat, we used the value of rotational relaxation time averaged over the Q range studied in the fitting of WASP data.

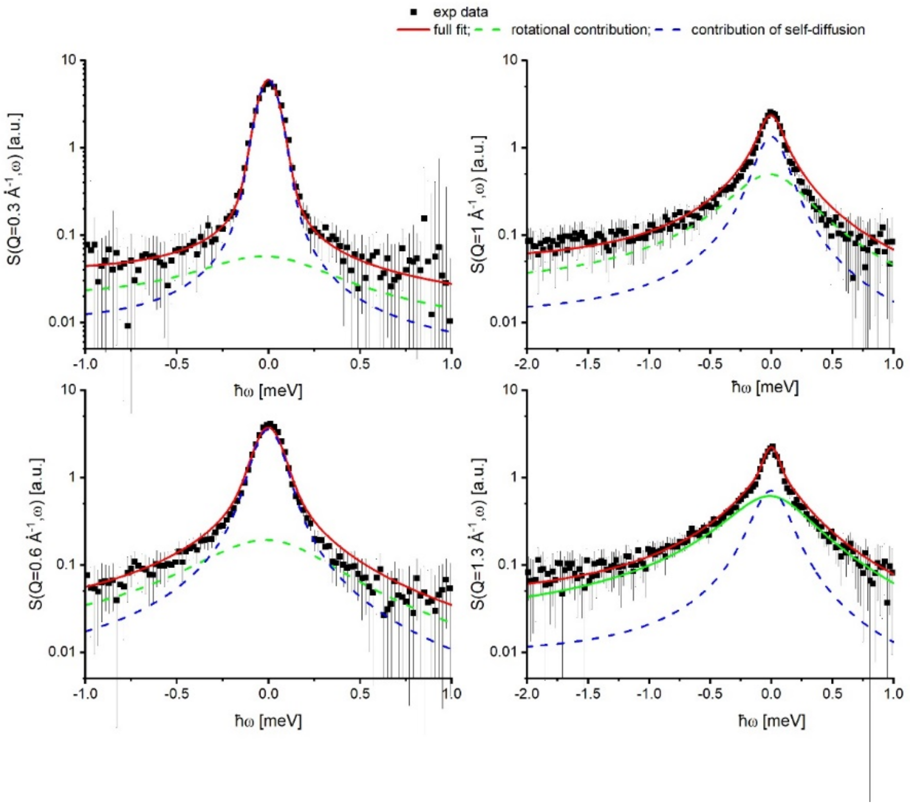

***Figure S1**. Incoherent dynamic structure factor at various Q (black squares) fitted by the model described in the text. The green dashed line indicates the contribution of a reorientational motion, the blue dashed line stands for the translational diffusive motion and the red line denotes the full model.*

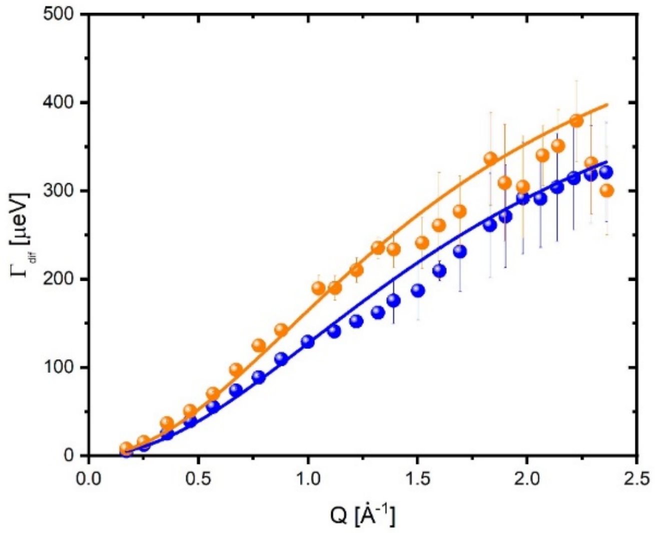

***Figure S2**. The broadening of the translational diffusive component fitted by the jump diffusion model according to the equations (s.17)-(s.20) at different temperatures: the blue points and line are the experimental data and fit at T=285 K; the orange points and line are the experimental data and fit at T=298 K. The fit allows us to establish the self-diffusion coefficients and residence times at corresponding temperatures: D_{self} =(1.69±0.07)x10^{-9} m^2/s and τ_0=1.94±0.0035 ps T=298 K at 285K and D_{self} =(2.23±0.024)x10^{-9} m^2/s and τ_0=1.70 ±0.002 ps at T=298 K .*

3. Experimental details for polarization analysis using Neutron Spin Echo spectrometer WASP

Neutron Spin Echo uses neutron spin to probe relaxation molecular dynamics at nanosecond time scale. Under application of magnetic fields with different directions polarized neutron spins undergo Larmor precession, whose angle depends on neutron velocity. The neutrons interact with a scattering sample where the velocity of the scattered neutrons can be changed due to the neutron – sample inelastic exchange. These changes are analyzed by making the scattered neutrons to undergo Larmor spin precessions in the reverse direction. If the sample scattering is purely elastic the polarization is completely restored, independently of the distribution of neutron velocities in the incoming beam. Inelastic exchange leads to the change of velocity of the scattered neutrons and hence to the differences of the Larmor presession angles and to loss of precessing polarization. The measured precessing beam polarization is proportional to the intermediate scattering function given by eq. (6) in the main text.

For our experiment we have used the wide angle neutron spin echo spectrometer WASP[10] build recently at Institute Laue-Langevin in Grenoble, France. The typical experimental setup of the NSE spectrometer consist of tuning the magnetic field setups and spin flippers, which allow the implementation of the polarization analysis. Polarization analysis can be used, to determine the spin flip (SF) and spin conserving (non-spin flip, NSF) components of the scattered beam. Similarly, to the polarization analysis described in the main text the coherent scattering contributes to NSF only, while incoherent scattering contributes to spin flip scattering and to the non-flip scattering (eqs. (2)-(3) in the main text). To determine these components, we measured the count rates of the fully polarized beam and fully depolarized beam without any other change to its intensity or angular distribution. It corresponds in the ideal case to the depolarized beam intensity N^A as the neutron count averaged over neutron spin directions (Fig.S3). Spin depolarization was achieved by using two 90° spin flippers, each of them were aimed to effectively "depolarize" the beam by turning the polarization direction perpendicular to the magnetic (guide) field direction independently from each other. This is a very efficient and safe method for pure beam depolarization and can be verified with a precision of 10^{-4} or better by the absence of any precessing beam polarization. Fully polarized beam intensity N^O has been measured without any spin turn or flip operation at the point "O", which actually corresponds to the theoretical maximum of a full NSE precession signal at the magnetic field value H_0 at the Fig.S3. The polarization efficiency *P* of the global instrument can be effectively measured for each detector independently using a calibration sample with well-known polarization behavior and is defined as

$$P = \frac{N^O - N^A}{N^A} \qquad \text{(s.21)}$$

N stands for observed counting rates at the corresponding points in Figure S3. *P* can be positive or negative, depending on the relative signs of the polarizer and analyzer actions. It is negative for WASP. For a sample with nuclear coherent and nuclear spin incoherent scattering, in view of the definition of P in eq. (s.21), we will have

$$N_{COH}^{O} = N_{COH}^{A}\,(1 + P) \qquad \text{(s.22)}$$

$$N_{INC}^{O} = N_{INC}^{A}\left(1 - \frac{P}{3}\right) \qquad \text{(s.23)}$$

Using these equations, we can determine the fractions of coherent scattering N_{COH}^{A} and incoherent scattering N_{INC}^{A}, integrated over the instrumental energy transfer window determined by the transmission of the analyzer system as:

$$N_{COH}^{A} = 0.75 * \frac{\left[N^O - N^A\left(1 - \frac{P}{3}\right)\right]}{P} \qquad \text{(s.24)}$$

$$N_{INC}^{A} = N^A - N_{COH}^{A} \qquad \text{(s.25)}$$

To verify the validity of this approach we have tested it on the TiZr calibration sample whose coherent cross section is negligible and the isotopic and spin incoherent scattering cross sections are well known. The results show very good agreement with below 1 % error for the determination of the components with different polarization behaviors in its scattering cross section.

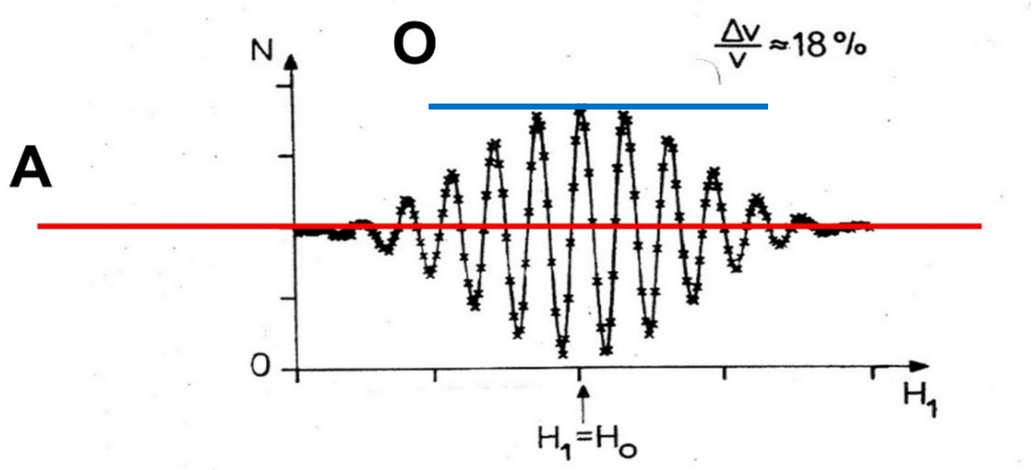

***Figure S3**. The scheme illustrating the measurement of the polarization at two different conditions. The dots indicate the measured echo signal at different values of magnetic field H. The position "O" shows the counting rate for the fully polarized beam, while the line marked as "A" indicates the fully depolarized beam.*

In the experiment we have used 4 Å incoming neutron wavelength which allowed us to cover Q range from 0.17 to 2.5 $Å^{-1}$ simultaneously. Additional scans were made at 6 Å incoming neutron wavelength to determine multiple scattering intensities and corresponding corrections. Samples were placed in Aluminum sample container with hollow cylindrical geometry with 0.1 mm and 0.05 mm thickness for H_2O and 0.4 mm for D_2O.

4. Multiple scattering corrections

The multiple scattering at 298 K was calculated according to the procedure described in the main text using the data at 4 and 6 Å with various sample thicknesses. Using this procedure, we have determined that for the samples with 0.1 mm thickness the multiple scattering contributes to the measured apparent coherent quasielastic structure factor by a Q independent 3.15 ± 0.07 % fraction of the incoherent single scattering structure factor averaged for Q < 1 $Å^{-1}$. Figure S4 shows measured and corrected coherent structure factors and demonstrates good agreement between the sets for different wavelengths and different sample thicknesses after the correction for the multiple scattering contribution. This confirms the validity of our model independent approach.

For the T=285 K only one set of data using 4 Å incoming neutron wavelength have been measured due to the beam time available. Based on the weak temperature dependence of the quasielastic structure factors we assumed the weak temperature dependence of the multiple scattering. Therefore, we used the procedure established for 298 K and corrected the coherent structure factor and NSE data for the same amount as for 298 K.

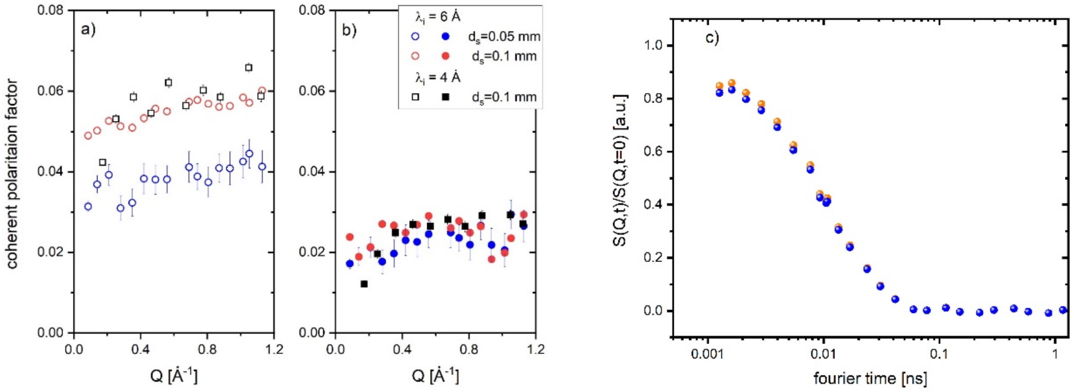

Figure S4 *Coherent quasielastic structure factors as measured (a) for different sample thicknesses and using various experimental setups, and (b) after correction for multiple scattering; (c) NSE spectra at Q = 0.58 Å⁻¹ and 298 K as measured (orange circles) and after correction for the multiple scattering contribution (blue circles).*

5. Temperature dependence of the relaxation times and additional details

The data corrected for multiple scattering were analysed using the model described in the main text. To reduce the influence of statistical fluctuations in the fit for 285 K we have used the average rotation times τ_{ROT}=1.38 ps deduced previously from NEAT data. The relaxation times obtained from WASP data for T=285 K are shown on the Figure S5a. Figure S5b shows the temperature dependence of the coherent relaxation times in H_2O and in D_2O as comparison. As it can be seen the coherent relaxation times at 285 K are slower compared to T=298 K and range from 2 to 3 ps. In addition, we observe a slight

increase of the coherent relaxation times around 1.5 Å, probably reflecting the strengthening of the short range order interactions.

In the final step we have fitted unpolarized H_2O spectra measured on NEAT with all three components. Results of the fit are shown on the Figure S6.

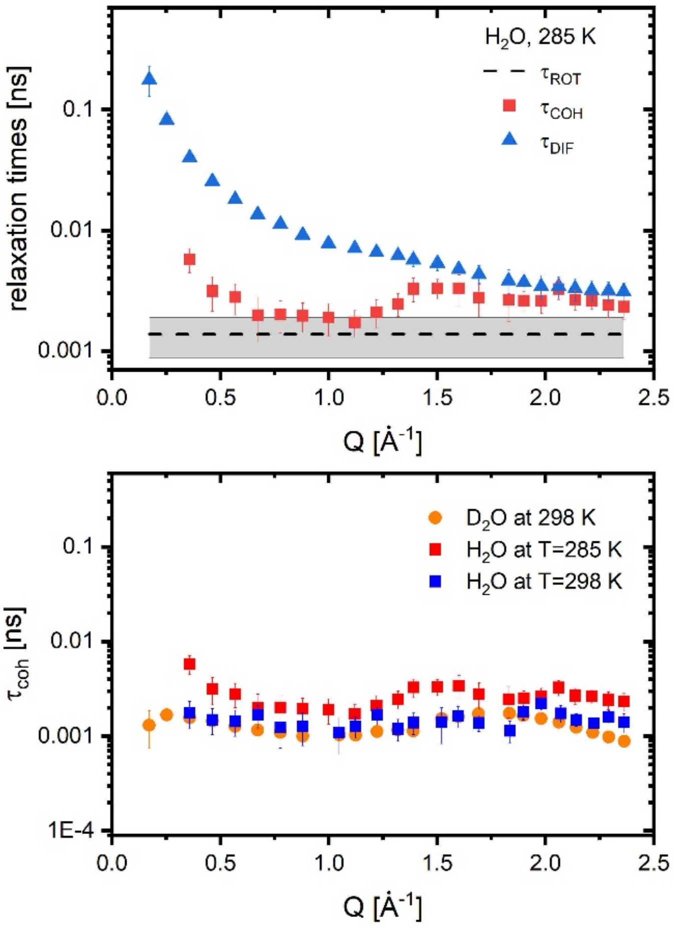

Figure S5. *a) Relaxation times for the reorientational, diffusive and coherent processes in H2O at 285 K. The dashed line represents the average value of the reorientational times determined by polarization analysis using NEAT. The gray area around the dashed line represents the statistical error range. b) Temperature dependence of the relaxation times of the coherent contribution in* H_2O *and* D_2O

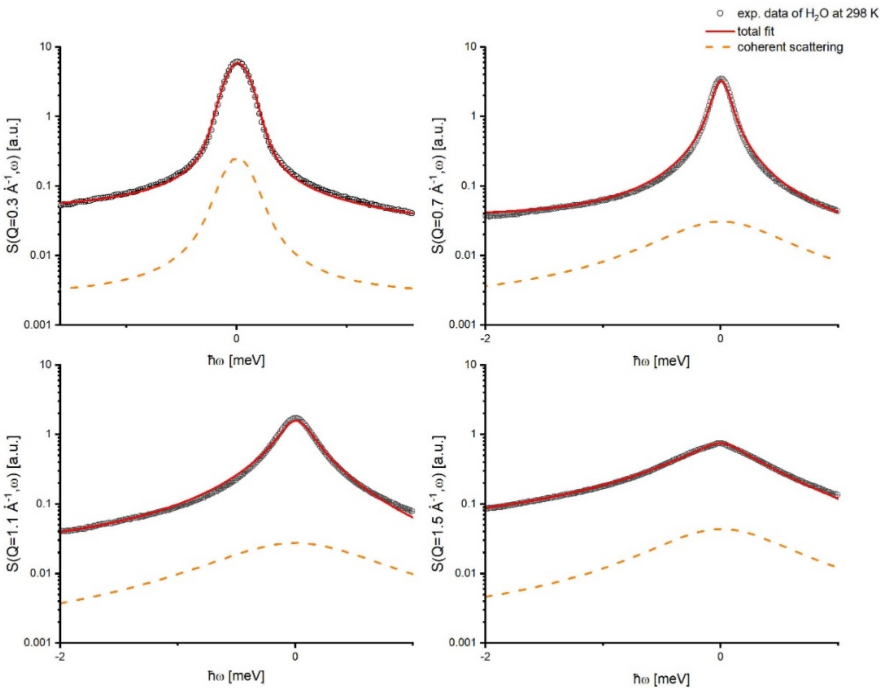

Figure S6. *Dynamic structure factors measured with unpolarized neutrons on spectrometer NEAT (hollow circles) at different Q values fitted by the model, which incorporates coherent and incoherent contributions.*

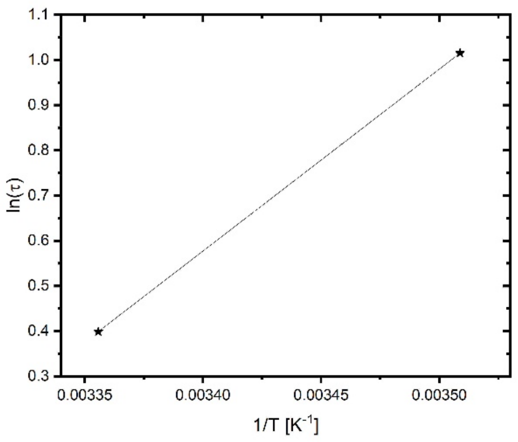

Figure S7. *Arrhenius plot of the relaxation time for the coherent process* τ_{COH} *measured at 298 K and 285 K (blue stars). The data are fitted with a linear function (black line), yielding an activation energy of 347 meV, corresponding to approximately 8 kcal/mol.*

References:

1. Squires GL. *Introduction to the theory of thermal neutron scattering*. Courier Corporation (1996).

2. Pynn R. Neutron scattering—a non-destructive microscope for seeing inside matter. In: *Neutron applications in earth, energy and environmental sciences* (ed^(eds). Springer (2009).

3. Gainov R, Mezei F, Füzi J, Russina M. Design concepts for a supermirror V-cavity based combined beam polarizer and compressor system for the upgraded neutron time-of-flight spectrometer NEAT. *Nuclear Instruments and Methods in Physics Research Section A: Accelerators, Spectrometers, Detectors and Associated Equipment* **930**, 42-48 (2019).

4. Günther G*, et al.* Polarized beam option for the time-of-flight spectrometer NEAT. In: *Journal of Physics: Conference Series* (ed^(eds). IOP Publishing (2019).

5. Babcock E, Mattauch S, Ioffe A. High level of 3He polarization maintained in an on-beam 3He spin filter using SEOP. *Nuclear Instruments and Methods in Physics Research Section A: Accelerators, Spectrometers, Detectors and Associated Equipment* **625**, 43-46 (2011).

6. Babcock E*, et al.* Recent on-beam tests of wide angle neutron polarization analysis with a 3He spin filter: Magic PASTIS on V20 at HZB. In: *Journal of Physics: Conference Series* (ed^(eds). IOP Publishing (2017).

7. Salhi Z*, et al.* First result from the magic-PASTIS using large 3He SEOP-polarized GE180 doughnut cell. In: *Journal of Physics: Conference Series* (ed^(eds). IOP Publishing (2016).

8. Arnold O*, et al.* Mantid—Data analysis and visualization package for neutron scattering and μ SR experiments. *Nuclear instruments and methods in physics research section a: accelerators, spectrometers, detectors and associated equipment* **764**, 156-166 (2014).

9. Teixeira J, Bellissent-Funel M, Chen SH, Dianoux AJ. Experimental determination of the nature of diffusive motions of water molecules at low temperatures. *Phys Rev A Gen Phys* **31**, 1913-1917 (1985).

10. Fouquet P, Ehlers G, Farago B, Pappas C, Mezei F. The wide-angle neutron spin echo spectrometer project WASP. *Journal of Neutron Research* **15**, 39-47 (2007).
